# Supplementary material for: Human-ignited fires result in more extreme fire behavior and ecosystem impacts
Source: Nat Commun. 2022 May 17;13:2717. doi: 10.1038/s41467-022-30030-2 (PMC9114381; doi:10.1038/s41467-022-30030-2)
Supplement: Supplementary file 1 — Supplementary Information [file 41467_2022_30030_MOESM1_ESM.pdf]

Supplementary Information for:

Human-ignited fires result in more extreme fire behavior and ecosystem impacts

Stijn Hantson[1,2*], Niels Andela[3,4], Michael L. Goulden[5], James T. Randerson[5]

[1] Geospatial Data Solutions Center, University of California, Irvine CA 92697, USA.

[2] Faculty of Natural Sciences, Universidad del Rosario, Bogotá, Colombia.

[3] School of Earth and Environmental Sciences, Cardiff University, Cardiff, Wales.

[4] Biospheric Sciences Laboratory, NASA Goddard Space Flight Center, Greenbelt, MD 20771, USA.

[5] Department of Earth System Science, University of California, Irvine, CA 92697, USA.

*Corresponding author:
Stijn Hantson
Email: stijn.hantson@urosario.edu.co
Tel: +57 3115901521

Supplementary Methods

Supplementary Methods 1: Validation of the daily fire growth database

We used daily fire growth of 14 fires (downloaded from: https://data-nifc.opendata.arcgis.com) with near daily fire size data collected as a way to evaluate our fire spread algorithm. These fires are of various size spread throughout the western US (Supplementary Table 3). We compare this to the MODIS burnt area product (MCD64A1 C6) [1] which has been used to extract daily fire spread rates [2]. On average, the temporal accuracy of the fire growth is better than that of MCD64 with a mean RMSE of 1.89 days for VIIRS compared to a RMSE of 2.49 for MCD64. However, most of the error are due to small mismatches in areas which burnt at very different times (e.g. when areas burnt at very different times near each other) which can lead to large differences in day of detection but have limited impact on our characterization of fire spread rates. Therefore, we analyzed the frequency distribution of daily fire expansion between the reference fires, the VIIRS fire spread and the amount of burnt area per day from MCD64 (Supplementary Figure 11). The frequency in growth rates between the reference and the VIIRS derived data are close to each other, while the MCD64 data shows a too high frequency of small fire expansions, and an underestimation of days where fire expansion was very large (> 3000 ha day^{-1}).

Supplementary Figures

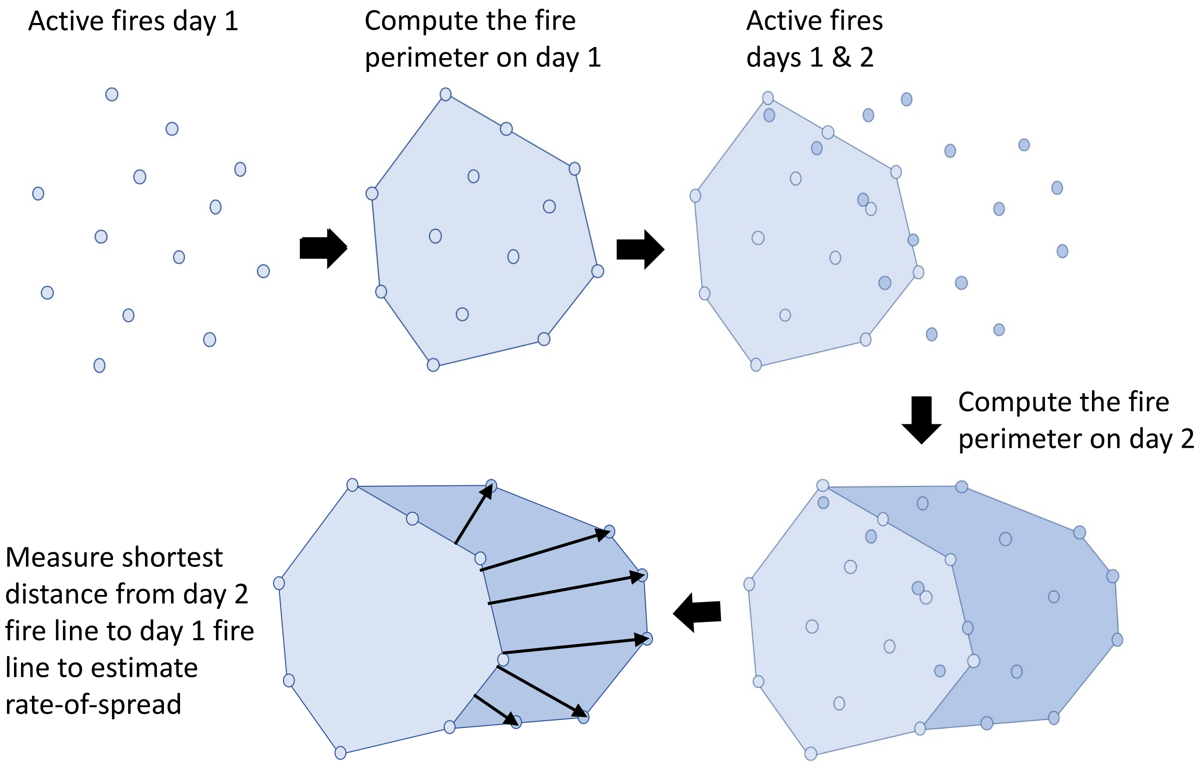

Supplementary Figure 1: Summary of how the daily rate-of-spread database was generated. Rate-of-spread is estimated for each active fire detected that day on the active fire line by estimating the minimum distance between the active fire and the previous day's fire perimeter outline. As a consequence, rate-of-spread cannot be estimated for the day of ignition. For our analysis, we estimate the daily rate-of-spread as the 95th percentile of spread rates estimated from the set of active fire detections along the fire perimeter on that day.

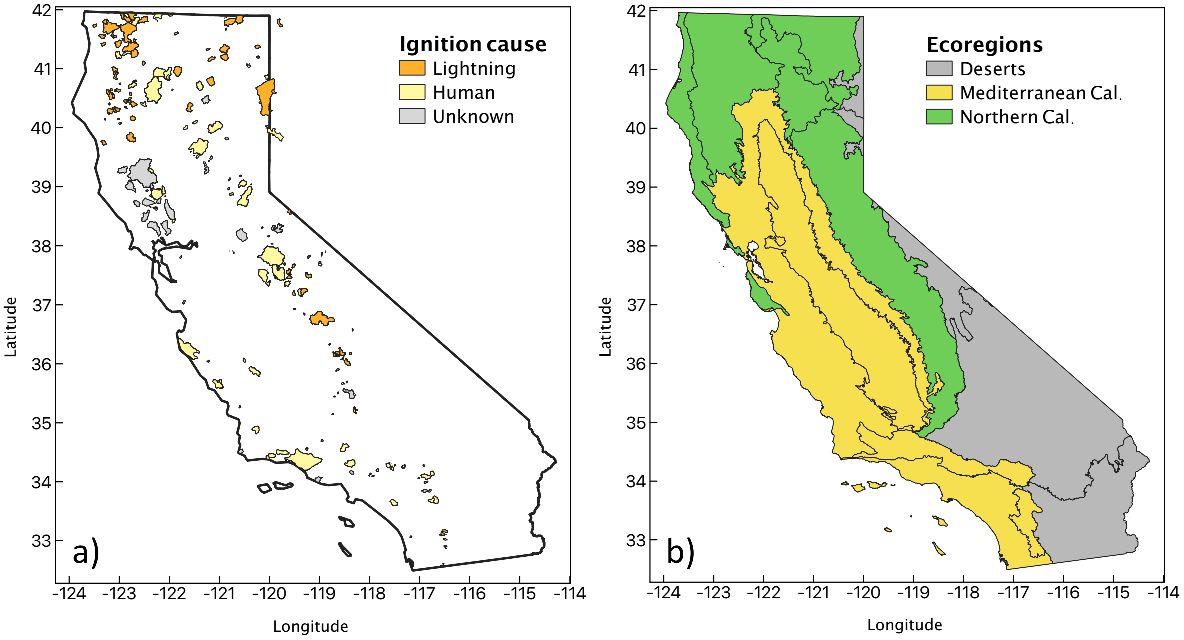

Supplementary Figure 2: a) Fires represented in the daily rate-of-spread database (2012-2018) across California, with the color of each fire indicating the cause of ignition. b) Distribution of ecoregions used in our analysis. The Northern California ecoregion combines the Marine west coast forest and Western cordillera level 2 ecoregions together. As few large fires take place in California desert ecosystems, we did not consider these areas specifically within in our analysis and have grouped cold and warm deserts together here.

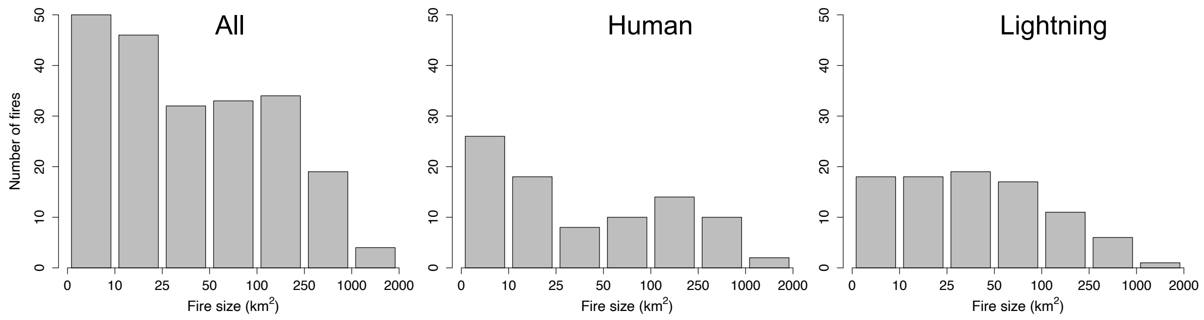

Supplementary Figure 3: Distribution of the final fire size represented in the daily rate-of-spread dataset for all fires in California, and those ignited by humans and lightning separately.

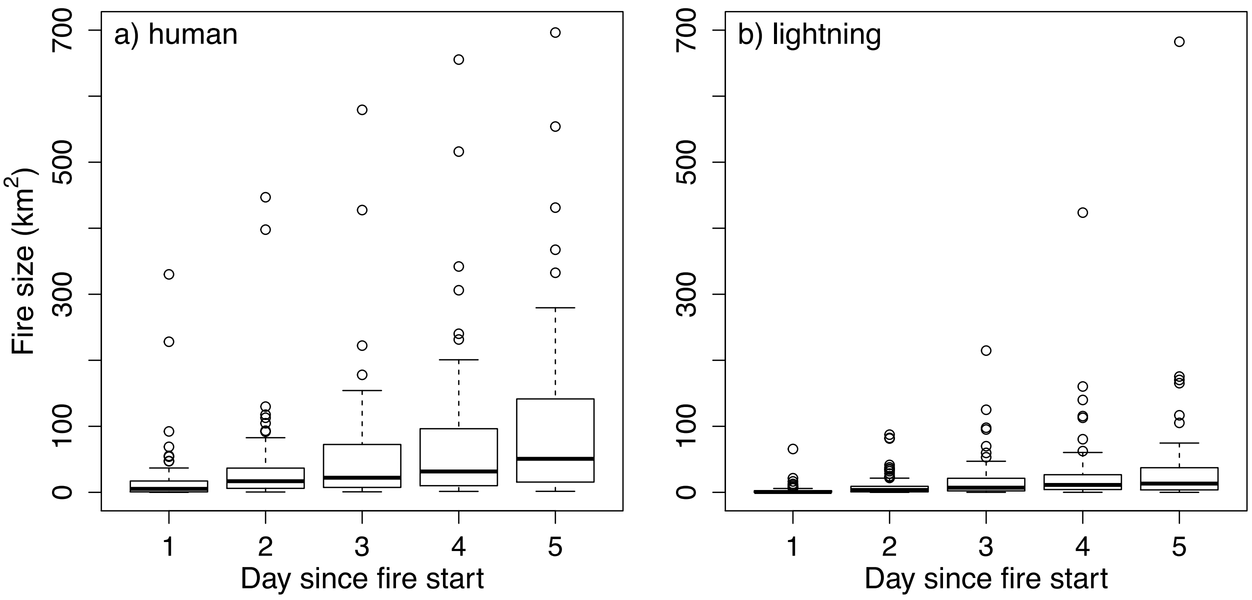

Supplementary Figure 4: Same as for Figure 1 but showing the full range of data including outliers. Boxplot of the fire size at the end of day 1 through day 5 after ignition for fires caused by humans (a) or lightning (b) in California. Differences between the two fire types are significant for each day (Welch Two Sample t-test, day 1: $t = 6.51$, $p = 8.27e^{-10}$; day 2: $t = 6.98$, $p = 1.20e^{-10}$; day 3: $t = 5.37$, $p = 3.29e^{-7}$; day 4: $t = 4.62$, $p = 9.47e^{-6}$; day 5: $t = 4.93$, $p = 3.089e^{-06}$). The sample size n= 82, 79, 67, 58, 44 from day 1-5 in panel a and n=90, 82, 77, 67, 64 from day 1-5 in panel b. The Boxplot represent the first quartile, the median, and the third quartile as a box, with the whiskers denote the minimum and maximum if within the range of the first quartile −1.5× the interquartile range and the third quartile +1.5× the interquartile range, while outliers are represented as points.

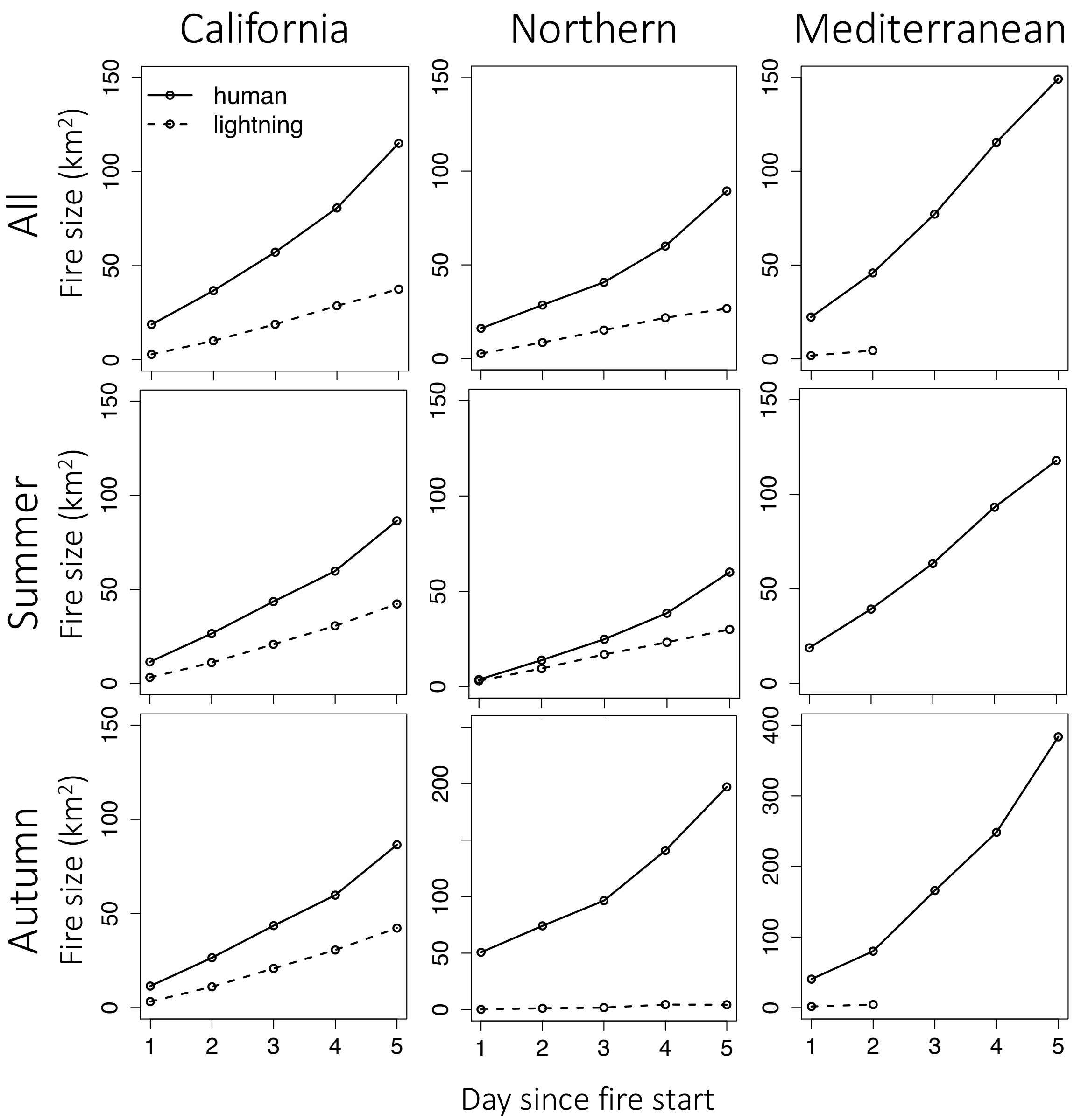

Supplementary Figure 5: Mean fire size at the end of day 1 through day 5 after ignition, for fires caused by humans (solid line) or lightning (dashed line) across California as a whole or for Northern or Mediterranean ecoregions. Fires are also separated by season in the different rows: all year is shown in the top row, summer (June, July, and August) is shown in the middle row, and autumn (September, October, and November) is shown in the bottom row.

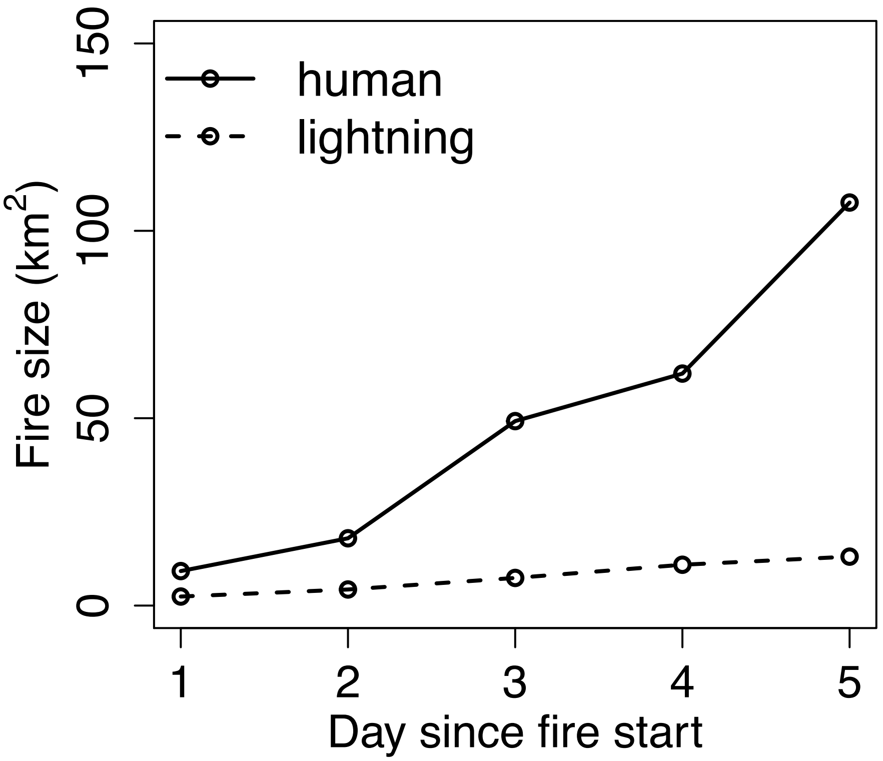

Supplementary Figure 6: Mean fire size for days 1-5 following ignition, for fires caused by humans (solid line) or lightning (dashed line) in the Northern ecoregion, specifically considering fires that had a mean aboveground biomass level greater than 150 Mg ha^{-1}.

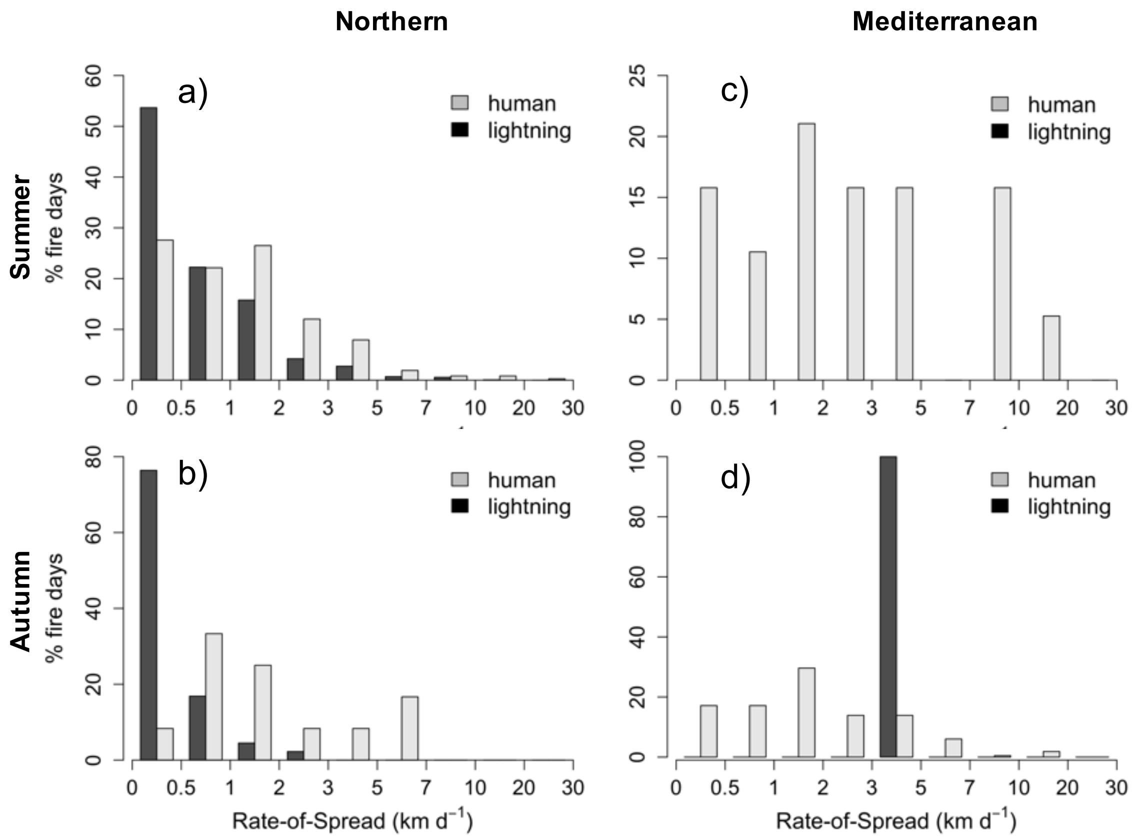

Supplementary Figure 7: Frequency distribution of the number of fire days with a 95th-percentile rate-of-spread separated by fire type across Northern California (a, b) and Mediterranean California (c, d) ecoregions and as a function of summer (May-august) (a, c) and autumn (b, d) periods. A fire day is a day on which active fire detections were present for a given fire after the first day of detection. Rate of spread is calculated as the 95th percentile of spread rates along the full active fire perimeter on that day.

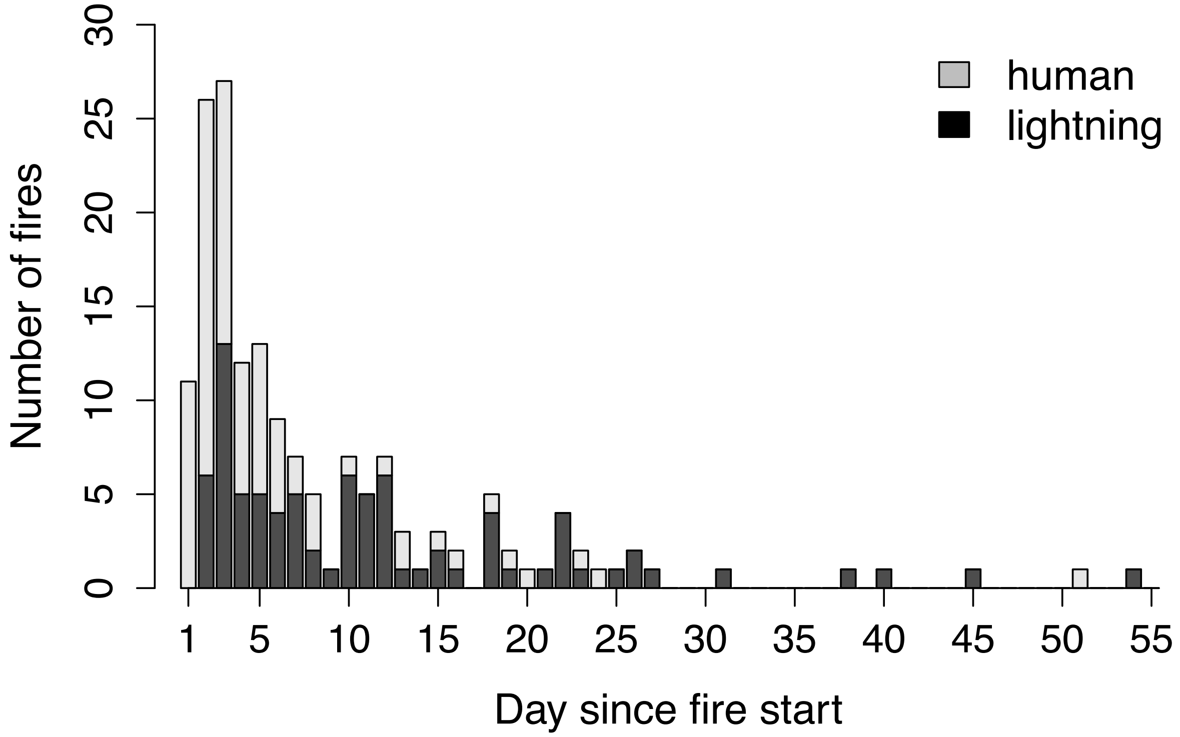

Supplementary Figure 8: The number of days it takes for fires in the rate-of-spread database to reach 75% of their final fire size for both human- and lightning-caused fires. The x-axis is cutoff at 55 days.

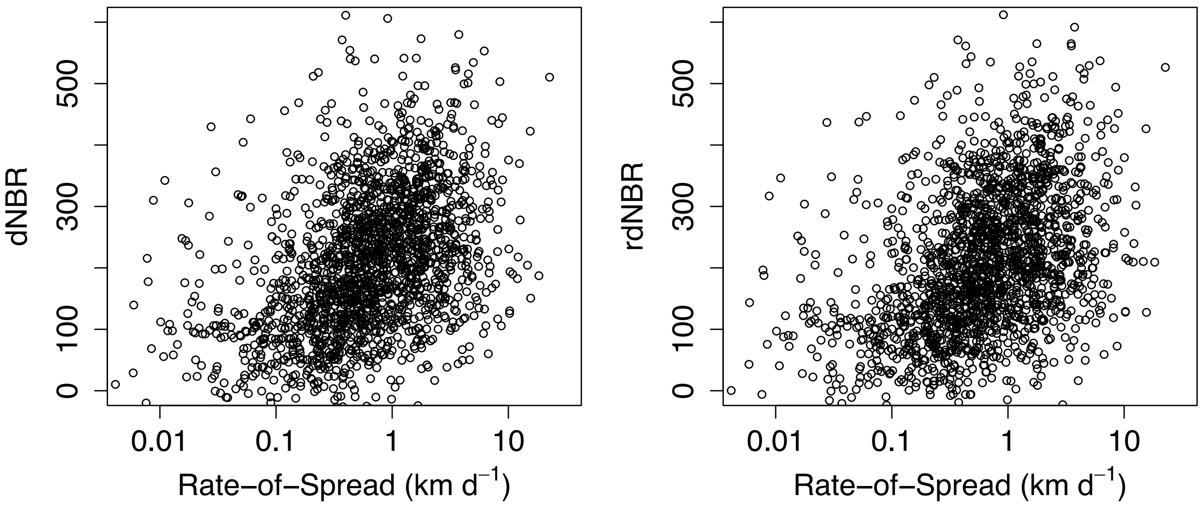

Supplementary Figure 9: Relation between daily fire rate-of-spread and fire severity, as represented by the difference normalized burn ratio (dNBR; F-test, F-statistic = 475.3, $R^2 = 0.22$, $p < 2.2e^{-16}$) and the relative difference normalized burn ratio (rdNBR; F-test, F-statistic = 433.5, $R^2 = 0.20$, $p < 2.2e^{-16}$). Higher values for both indices indicate higher fire severity. Rate-of-spread is calculated as the 95th percentile of fire spread rates along the full active fire perimeter fire on that day.

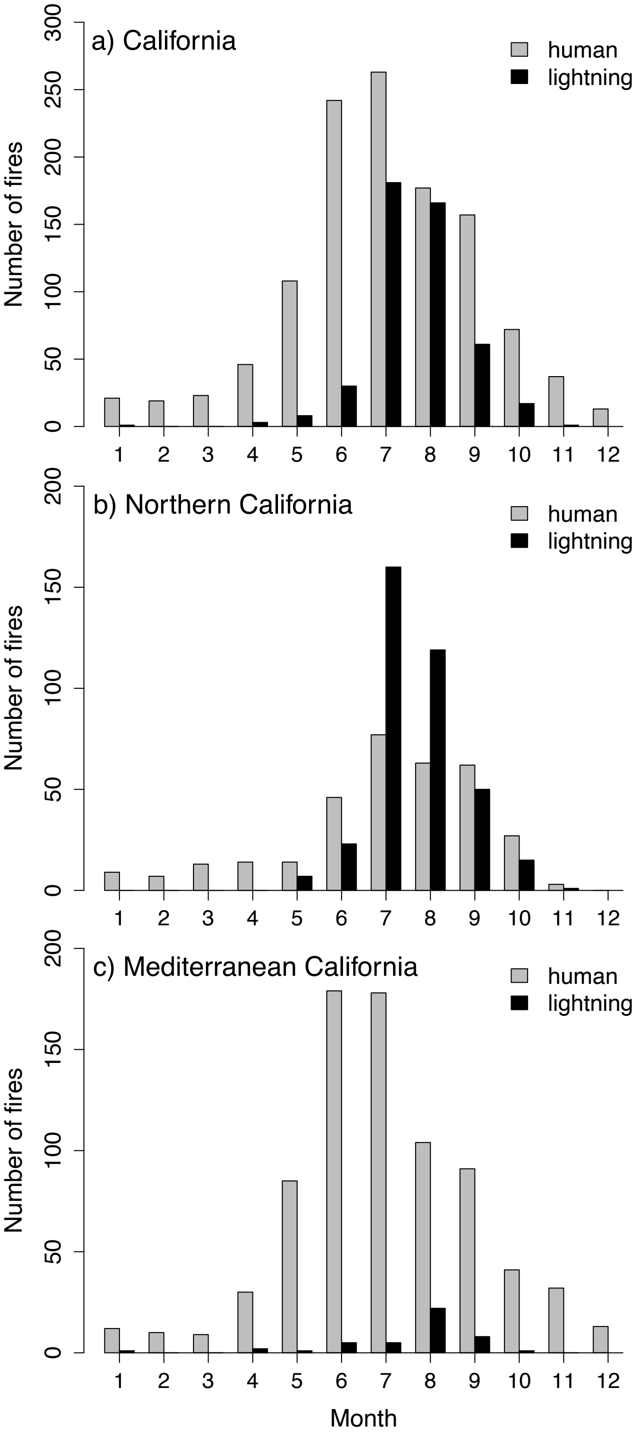

Supplementary Figure 10: Frequency distribution by month of the number of human-caused and lightning-caused fires across a) California, b) Northern California and c) Mediterranean California.

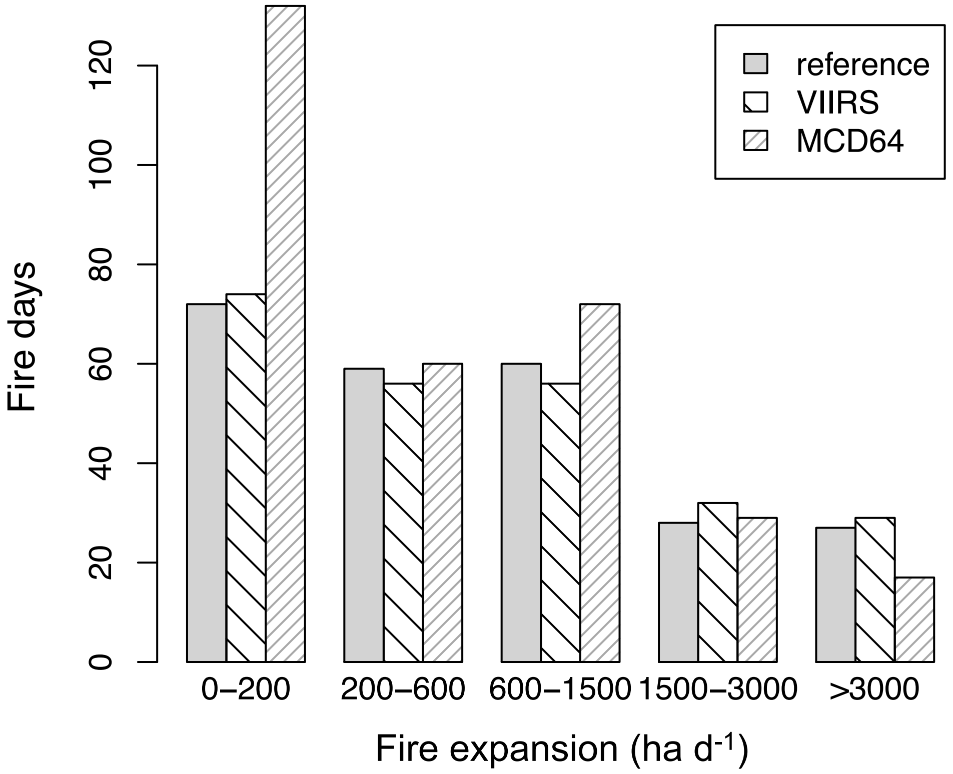

Supplementary Figure 11: Frequency distribution of daily fire expansion for a set of 14 individual fires from the forest service and as extracted from VIIRS thermal anomalies and MODIS burned area (MCD64A1). Days without reference values were excluded, as well as the day afterwards to characterize daily fire growth values correctly.

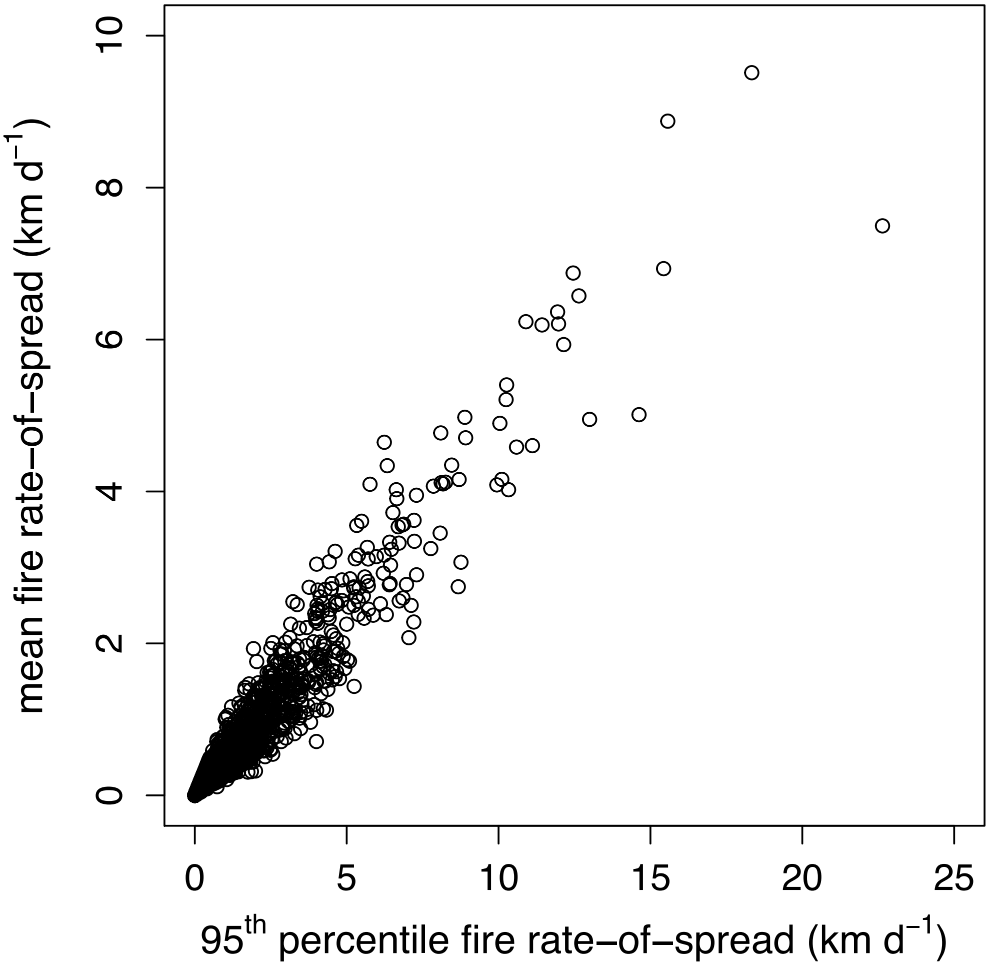

Supplementary Figure 12: Relation between the 95th percentile of the spread rates from the active fire detections along the fire line at the end of that day and the mean fire rate-of-spread.

Supplementary Tables

Supplementary Table 1: Mean environmental conditions on the ignition day for human- and lightning-caused fires in FRAP across all of California and for the Northern California (2012-2018). Significant differences for human- and lightning-caused fires were assessed using the Welch Two Sample t-test. The comparisons here are for the complete FRAP dataset, representing a larger set of fires than in the fire rate-of-spread dataset.

	California			Northern California		
	lightning	human	p	lightning	human	p
Fire number	486	1196	-	375	335	-
Mean fire size (km^2)	17.0	9.7	0.08	16.6	15.2	0.81
burn index (-)	32.8	**53.6**	$<2.2e^{-16}$	31.6	**52.0**	$<2.2e^{-16}$
energy release component (-)	64.4	**67.6**	$1.3e^{-4}$	63.5	**67.8**	$1.2e^{-3}$
potential evapotranspiration (mm d^{-1})	7.6	**8.6**	$3.7e^{-16}$	7.4	**7.9**	$2.3e^{-3}$
fm100 (%)	8.2	8.4	0.24	8.2	8.2	0.79
fm1000 (%)	**8.7**	9.8	$<2.2e^{-16}$	**8.8**	9.9	$1.1e^{-5}$
Precipitation (mm d^{-1})	1.3	**0.2**	$5.6e^{-12}$	1.4	**0.6**	$9.7e^{-3}$
maximum relative humidity (%)	59.0	61.4	0.03	58.4	58.0	0.82
minimum relative humidity (%)	22.9	**19.4**	$3.0e^{-8}$	23.5	**19.8**	$3.2e^{-5}$
minimum temperature (K)	286.2	286.0	0.39	**285.5**	283.7	$2.1e^{-6}$
maximum temperature (K)	302.2	303.2	0.01	301.4	300.9	0.39
vapor pressure deficit (kPa)	2.0	2.1	0.09	1.9	1.9	0.49
windspeed (m/s)	2.6	**3.3**	$<2.2e^{-16}$	2.5	**3.3**	$1.7e^{-14}$
Aboveground biomass (Mg/ha)	131	39	$<2.2e^{-16}$	159	99	$4.0e^{-13}$
forest cover (%)	63	25	$<2.2e^{-16}$	73	56	$7.2e^{-4}$

Bold values indicate the highest fire risk for each meteorological variable with a significant difference between the two fire types ($p<0.01$).

Supplementary Table 2: Mean environmental conditions on the ignition day for human- and lightning-caused fires in FRAP across Mediterranean California and for the Northern California summer fires (2012-2018). Significant differences for human- and lightning-caused fires were assessed using the Welch Two Sample t-test. The comparisons here are for the complete FRAP dataset, representing a larger set of fires than in the fire rate-of-spread dataset.

	Mediterranean California			Northern California		
	lightning	human	p	lightning	human	p
Fire number	45	784	-	352	248	-
Mean fire size (km^2)	2.0	7.1	0.03	17.4	17.9	0.95
burn index (-)	34.3	**53.5**	$4.1e^{-5}$	32.1	**56.3**	$<2.2e^{-16}$
energy release component (-)	63.2	66.7	0.17	64.5	**74.3**	$2.0e^{-15}$
potential evapotranspiration (mm d^{-1})	8.5	8.9	0.26	7.6	**8.7**	$2.8e^{-12}$
fm100 (%)	9.0	8.6	0.48	8.0	**7.3**	$1.1e^{-3}$
fm1000 (%)	**8.7**	9.9	$2.3e^{-3}$	8.7	8.5	0.23
Precipitation (mm d^{-1})	1.3	**0.1**	$4.9e^{-4}$	1.3	**0.1**	$1.1e^{-10}$
maximum relative humidity (%)	66.5	63.9	0.33	57.3	56.0	0.43
minimum relative humidity (%)	23.9	**19.6**	0.01	22.8	**18.4**	$7.3e^{-7}$
minimum temperature (K)	**290.4**	287.0	$9.6e^{-6}$	286.1	285.7	0.26
maximum temperature (K)	305.8	304.1	0.06	302.2	**303.7**	$2.0e^{-3}$
vapor pressure deficit (kPa)	2.3	2.2	0.38	2.0	2.2	0.03
windspeed (m/s)	3.0	**3.3**	0.01	2.5	**3.1**	$1.3e^{-9}$
Aboveground biomass (Mg/ha)	17	16	0.73	155	98	$2.3e^{-10}$
forest cover (%)	39	14	$7.2e^{-5}$	72	58	0.01

Bold values indicate the highest fire risk for each meteorological variable with a significant difference between the two fire types ($p<0.01$).

Supplementary Table 3: Summary statistics of the 14 reference fires used to validate the VIIRS-based fire rate-of-spread algorithm. RMSE statistics are indicate the difference in days between the reference day burned and that as extracted from the rate-of-spread algorithm. We also show the difference between the reference data set and the timing of MODIS MCD64 burned area for comparison.

Fire name	Year	State	Area (ha)	VIIRS RMSE (days)	MCD64 RMSE (days)
Bagely Complex	2012	California	18620	1.720	2.529
Erskine	2016	California	19430	1.027	1.076
Fort Complex	2012	California	9570	3.755	3.877
Gladiator	2012	Arizona	6570	1.079	1.425
Lake	2015	California	12690	2.176	2.815
Little Sand	2012	Colorado	10080	3.937	3.578
North Pass	2012	California	16990	1.317	2.890
Poco	2012	Arizona	4840	0.925	2.564
Powerhouse	2013	California	12250	0.672	1.061
Reading	2012	California	11360	1.723	3.232
Rough	2015	California	61360	4.249	3.767
Sunflower	2012	Arizona	7060	1.105	1.336
Waldo Canyon	2012	Colorado	7384	1.227	3.568
Whitewater Baldy	2012	New Mexico	117148	2.070	2.143
		Mean	33488	1.890	2.491

Supplementary References

1 Giglio, L., Boschetti, L., Roy, D. P., Humber, M. L. & Justice, C. O. The Collection 6 MODIS burned area mapping algorithm and product. *Remote Sensing of Environment* **217**, 72-85, doi:https://doi.org/10.1016/j.rse.2018.08.005 (2018).

2 Andela, N. *et al.* The Global Fire Atlas of individual fire size, duration, speed and direction. *Earth Syst. Sci. Data* **11**, 529-552, doi:10.5194/essd-11-529-2019 (2019).
