# Supplementary material for: Human-ignited fires result in more extreme fire behavior and ecosystem impacts
Source: Nat Commun. 2022 May 17;13:2717. doi: 10.1038/s41467-022-30030-2 (PMC9114381; doi:10.1038/s41467-022-30030-2)
Supplement: Supplementary file 3 — Reporting Summary [file 41467_2022_30030_MOESM3_ESM.pdf]

nature portfolio

Corresponding author(s): Stijn Hantson

Last updated by author(s): Jan 23, 2022

Reporting Summary

Nature Portfolio wishes to improve the reproducibility of the work that we publish. This form provides structure for consistency and transparency in reporting. For further information on Nature Portfolio policies, see our Editorial Policies and the Editorial Policy Checklist.

Statistics

For all statistical analyses, confirm that the following items are present in the figure legend, table legend, main text, or Methods section.

n/a	Confirmed	
☐	☒	The exact sample size (*n*) for each experimental group/condition, given as a discrete number and unit of measurement
☒	☐	A statement on whether measurements were taken from distinct samples or whether the same sample was measured repeatedly
☐	☒	The statistical test(s) used AND whether they are one- or two-sided<br>*Only common tests should be described solely by name; describe more complex techniques in the Methods section.*
☒	☐	A description of all covariates tested
☒	☐	A description of any assumptions or corrections, such as tests of normality and adjustment for multiple comparisons
☐	☒	A full description of the statistical parameters including central tendency (e.g. means) or other basic estimates (e.g. regression coefficient) AND variation (e.g. standard deviation) or associated estimates of uncertainty (e.g. confidence intervals)
☐	☒	For null hypothesis testing, the test statistic (e.g. F, t, r) with confidence intervals, effect sizes, degrees of freedom and P value noted<br>*Give P values as exact values whenever suitable.*
☒	☐	For Bayesian analysis, information on the choice of priors and Markov chain Monte Carlo settings
☒	☐	For hierarchical and complex designs, identification of the appropriate level for tests and full reporting of outcomes
☒	☐	Estimates of effect sizes (e.g. Cohen's *d*, Pearson's *r*), indicating how they were calculated

Our web collection on statistics for biologists contains articles on many of the points above.

Software and code

Policy information about availability of computer code

Data collection	All data processing and analysis was performed with R 4.0.3. Packages used are : maptools_1.1-2; raster_3.5-2; alphahull_2.2 ; rgdal_1.5-27; geosphere_1.5-14; rgeos_0.5-8; sp_1.4-6; igraph_1.2.9; foreach_1.5.1; doParallel_1.0.16; png_0.1-7. The code to generate the fire growth dataset can be found here: https://doi.org/10.5281/zenodo.6362832
Data analysis	All data processing and analysis was performed with R 4.0.3. Packages used are : maptools_1.1-2; raster_3.5-2; alphahull_2.2 ; rgdal_1.5-27; geosphere_1.5-14; rgeos_0.5-8; sp_1.4-6; igraph_1.2.9; foreach_1.5.1; doParallel_1.0.16; png_0.1-7.

For manuscripts utilizing custom algorithms or software that are central to the research but not yet described in published literature, software must be made available to editors and reviewers. We strongly encourage code deposition in a community repository (e.g. GitHub). See the Nature Portfolio guidelines for submitting code & software for further information.

Data

Policy information about availability of data

All manuscripts must include a data availability statement. This statement should provide the following information, where applicable:

- Accession codes, unique identifiers, or web links for publicly available datasets
- A description of any restrictions on data availability
- For clinical datasets or third party data, please ensure that the statement adheres to our policy

The California fire growth dataset generated in this study has been deposited under accession code http://doi.org/10.5281/zenodo.4248662 and will be updated regularly. The FRAP fire perimeters are available at https://frap.fire.ca.gov/frap-projects/fire-perimeters/. The GridMET climate dataset is available from http://www.climatologylab.org/gridmet.html. The tree mortality product generated by the Forest Service 39 is available at: https://www.fs.usda.gov/detail/r5/landmanagement/gis/?cid=STELPRDB5327833. The vegetation biomass dataset from Oregon State University Landscape Ecology, Modeling, Mapping & Analysis

March 2021

(LEMMA) is available at: https://lemmadownload.forestry.oregonstate.edu. The MTBS fire severity data is available at: https://www.mtbs.gov.

Field-specific reporting

Please select the one below that is the best fit for your research. If you are not sure, read the appropriate sections before making your selection.

☐ Life sciences ☐ Behavioural & social sciences ☒ Ecological, evolutionary & environmental sciences

For a reference copy of the document with all sections, see nature.com/documents/nr-reporting-summary-flat.pdf

Ecological, evolutionary & environmental sciences study design

All studies must disclose on these points even when the disclosure is negative.

Study description	We study how timing and location of fire ignitions influence fire rate of spread and ecosystem impact across california
Research sample	All large, multi day fires between 2012-2018 for which daily fire spread could be estimated. Only multi-day fires are considered as fire rate of spread can only be estimated from multi-day fires. The time period is limited bu the availability of VIIRS active fire data, with the satellite being launched towards the end of 2011. The main dataset provides daily fire growth data and was produced by the authors of this study and can be accessed here: http://doi.org/10.5281/zenodo.4248662
Sampling strategy	All available data was used.
Data collection	We use active fire detections from the VIIRS instrument onboard the Suomi National Polar-orbiting Partnership (Suomi-NPP) satellite which provided data at 12h intervals. Active fire detections were merged with the California reference burned area dataset (FRAP) and daily fire progression was generated for each fire day for which active fire detections were available.
Timing and spatial scale	2012-2018 across California, with the original active fire detections having a 375 m spatial resolution at nadir.
Data exclusions	no data were excluded
Reproducibility	All results are reproducible
Randomization	no different treatment groups are used, so randomization is not relevant for our study.
Blinding	This is not relevant for our research, as there is no indication of existing biases which could have influenced data acquisition and/or analysis.

Did the study involve field work? ☐ Yes ☒ No

Reporting for specific materials, systems and methods

We require information from authors about some types of materials, experimental systems and methods used in many studies. Here, indicate whether each material, system or method listed is relevant to your study. If you are not sure if a list item applies to your research, read the appropriate section before selecting a response.

Materials & experimental systems

n/a	Involved in the study	
☒	☐	Antibodies
☒	☐	Eukaryotic cell lines
☒	☐	Palaeontology and archaeology
☒	☐	Animals and other organisms
☒	☐	Human research participants
☒	☐	Clinical data
☒	☐	Dual use research of concern

Methods

n/a	Involved in the study	
☒	☐	ChIP-seq
☒	☐	Flow cytometry
☒	☐	MRI-based neuroimaging
